# The predictive model for risk of chemotherapy-induced thrombocytopenia based on antineoplastic drugs for solid tumors in eastern China

**DOI:** 10.1038/s41598-023-27824-9

**Published:** 2023-02-23

**Authors:** Shishi Zhou, Bingxin Song, Chenghui Li, Wanfen Tang, Xia Zhang, Xiayun Jin, Xifeng Xu, Qinghua Wang, Hongjuan Zheng, Jianfei Fu

**Affiliations:** 1 Department of Medical Oncology, Jinhua Hospital, Zhejiang University School of Medicine, 351 Mingyue Road, Jinhua, 321000 Zhejiang Province China; 2 Zhejiang University School of Medicine, 866 Yuhangtang Road, Xihu District, Hangzhou, 310058 Zhejiang Province China

**Keywords:** Cancer, Medical research, Oncology, Risk factors

## Abstract

Chemotherapy-related thrombocytopenia (CIT) is a significant adverse event during chemotherapy, which can lead to reduced relative dose intensity, increased risk of serious bleeding and additional medical expenditure. Herein, we aimed to develop and validate a predictive nomogram model for prediction of CIT in patients with solid tumor. From Jun 1, 2018 to Sep 9, 2021, a total of 1541 patients who received 5750 cycles of chemotherapy were retrospectively enrolled. Cox regression analysis was performed to identify predictive factors to establish the nomogram model for CIT. The incidence of chemotherapy-induced thrombocytopenia was 21.03% for patient-based and 10.26% for cycles of chemotherapy. The top five solid tumors with CIT are cervix, gastric, bladder, biliary systemic, and ovarian. The incidence of chemotherapy dose delays in any cycle because of CIT was 5.39%. Multivariate analysis showed that tumor site, treatment line, AST, oxaliplatin, and capecitabine were significantly associated with CIT. Moreover, we established a nomogram model for CIT probability prediction, and the model was well calibrated (Hosme-Lemeshow *P* = 0.230) and the area under the receiver operating characteristic curve was 0.844 (Sensitivity was 0.625, Specificity was 0.901). We developed a predictive model for chemotherapy-induced thrombocytopenia based on readily available and easily assessable clinical characteristics. The predictive model based on clinical and laboratory indices represents a promising tool in the prediction of CIT, which might complement the clinical management of thrombocytopenia.

## Introduction

Cancer is a major public health problem worldwide. In 2021, 1,898,160 new cancer cases and 608,570 cancer deaths are projected to occur in the world^1^. Chemotherapy is still one of the main means of treatment for patients with locally advanced or metastatic cancer. Chemotherapy-induced thrombocytopenia (CIT) is defined as a peripheral platelet count less than 100 × 10^9^/L, with or without bleeding in cancer patients receiving myelosuppressive chemotherapy^2^. Prior studies estimated that more than 30% of patients with a solid tumor experienced thrombocytopenia^3,4^, of whom over 10% were Grade II and above^5^.CIT is a significant medical problem during chemotherapy, and it can lead to chemotherapy dose delays, dose reductions, and increased risk of serious bleeding events and additional medical expenditure^6,7^. In addition, multiple studies suggests that patients who receive lower chemotherapy dose intensity than planned experience worse overall survival (OS), progression-free survival (PFS) and disease-free survival than those receiving standard or dose-dense chemotherapy^4,8–10^.

Until now, the treatment options of CIT are still limited. Patients with platelet transfusion may develop allergic, febrile reactions and acquire infectious diseases^11,12^. Recombinant human interleukin-11 (rhIL-11) can either directly or indirectly induce cardiotoxicity^13,14^. The incidence of atrial fibrillation induced by rhIL-11 was about 0.49%^15^. And there has also been a case report of heart failure caused by rhIL-11^16^. So, rhIL-11 should be used cautiously when patients have congestive heart failure and atrial arrhythmia. The ecombinant human thrombopoietin (rhTPO) is the only thrombopoietin receptor agonist (TPO–RA) that receives market approval in China for the treatment of CIT. But repeated use of rhTPO can easily cross-react with endogenous TPO to produce anti-drug antibodies^18^. It seriously hinders the smooth implementation of anti-tumor therapy.

As is known to us all, the incidence and degree of CIT are various in patients with different tumors receiving diverse chemotherapy regimens. However, the data of CIT from clinical trials usually do not reflect the actual proportion of chemotherapy dose delays or dose reductions resulted from thrombocytopenia alone. In addition, patients with severe thrombocytopenia may be withdrawn from the clinical trial, which means that the relationship between CIT and survival cannot be confirmed experimentally. Thus, the clinical trial data is not a perfect substitute for the real- world setting. According to the actual condition and willingness of patients, the Real-World Study (RWS) focused on significant endpoint and conducted long-term follow-up in a large population. The incidence of malignancies is regionally characteristic, but some CIT-related RWS were not regionally generalized^3,4,7,19,20^, and should always be taken into consideration in geographic differences of CIT. In addition, some studies were aimed at clarifying the relationship between CIT and demographic characteristics and treatments, identifying CIT high-risk population and establishing CIT prediction model to guide clinical treatment in turn. However, the inclusion of risk factors was relatively simple^21–23^.

In the present study, we established a CIT prediction model based on the evaluation of the value of tumor site, treatment regimens, treatment line, TBIL level, AST level, hemoglobin and platelet counts, it will be a viable alternative to clinical diagnosis and therapy.

## Methods and materials

### Patients and clinical data collection

A retrospective cohort consisting of patients who received chemotherapy for solid tumour treatment, was selected from among all patients who received anti-tumor treatment in the Oncology Department of Jinhua Municipal Central Hospital from June, 2018 to September, 2021. In this study, the primary endpoint was a predictive model for chemotherapy-induced thrombocytopenia.

The inclusion criteria were as following: (1) Histologically or cytologically confirmed solid tumors were enrolled. (2) All patients were followed during all chemotherapy cycles. (3) All enrolled patients had at least one platelet count after the first occurrence of CIT. The exclusion criterions were as following: (1) Exclusion criteria included a history of hematologic malignancy(Excluding lymphoma), confirmed by fluorescence in situ hybridization (FISH) from bone marrow aspirate and biopsy or peripheral blood test in the prior 3 months. (2) A history of a prior symptomatic venous thromboembolic event (VTE) or arterial event. (3) Other reasons for exclusion included any serious concomitant medical condition that could interfere with the conduct of the clinical trial, such as unstable angina, renal failure requiring hemodialysis, or active infection requiring intravenous antibiotics. Tumors with less than 30 patients were classified as “Other tumor site”, and those with 2 or more primary sites were defined as multiple primary cancers(MPC). This study complies with the ethical standards of the Institutional Research Council and the Declaration of Helsinki. The occurrence of CIT(a platelet count < 100,000/ml) during all chemotherapy cycles was considered as the study outcome. Contrary to the traditional definition, we defined the first anticancer treatment at our center as first-line chemotherapy, and so on. To identify the CIT, a platelet count test was performed at the beginning of every chemotherapy cycle. Then, medical records of patients and hospital information system were reviewed.

### Study variables and outcome data assays

Medical records of solid tumor patients and hospital information system (the Haitai electronic case system and the Guide Patients Support Care (GPS) information system) were reviewed. The following parameters and measurement methods were used throughout the study.

### Variables definitions

#### Laboratory indicators

The clinically relevant CIT was defined as a platelet (PLT) count < 100,000/ml. The grades of thrombocytopenia were divided into grade 1 to 4. Respectively, 75,000/ml ≤ PLT < 100,000/ml was considered as grade 1, 50,000/ml ≤ PLT < 75,000/ml as grade 2, 25,000/ml ≤ PLT < 50,000/ml as grade 3, and PLT < 25,000/ml as grade 4. The definition of anemia was hemoglobin (HB) concentration < 110 g/L. The definition of leucopenia was white blood cell (WBC) count < 4,000/ml. The C-reactive protein (CRP) level > 8 mg/dL was considered as an increase in CRP. A total bilirubin (TBIL) level < 25 umol/L was considered as a decrease in bilirubin. Albumin (ALB) level < 30 g/L was considered as hypoalbuminemia. An increase in alanine aminotransferase was defined as alanine aminotransferase (ALT) level > 50 U/L and an increase in aspartate aminotransferase (AST) was defined as aspartate aminotransferase level > 35U/L.

#### Nutrition assessment tools

Body mass index (BMI, classified based on World Health Organization criteria), Patient-Generated Subjective Global Assessment (PG–SGA) score and Nutritional Risk Screening 2002 (NRS2002) score. Severe malnutrition was defined as body mass index (BMI) < 18.5 kg/m2. The final score of NRS2002 ranges from 0 to 7, with a score of ≥ 3 indicating a high nutritional risk. Based on the above assessments, patients are classified as well-nourished (PG–SGA A), moderately or suspected of being malnourished (PG–SGA B), or severely malnourished (PG–SGA C). PGSGA score < 4 was defined as PG–SGA A, 4 ≤ PGSGA score < 8 was defined as PG–SGA B and PGSGA score ≥ 8 defined as PG–SGA C.

#### Treatment line

The first time a patient came to our center for antineoplastic therapy was defined as first-line therapy, which was different from the traditional definition. It is defined as a line change treatment when the difference between treatment plans is greater than 50%. If the treatment plan was a line change treatment compared with the last treatment, the treatment line this time was defined as the original number of treatment line plus one.

#### Type of antineoplastic drugs

Typical Alkylating agents(cyclophosphamide, iso-cyclophosphamide, etc.), Platinum compounds(cisplatin, carboplatin, oxaliplatin, etc.), Antimetabolites(fluorouracil, capecitabine, S-1, TAS102, gemcitabine, pemetrexed), Plant alkaloids(taxane, vinorelbine, irinotecan, etoposide, etc.), Cytotoxic antibiotics and related substances(anthracycline, etc.), Hormones(tamoxifen, letrozole, fulvestrant, etc.), Immune Checkpoint Inhibitors (ICIs)(pembrolizumab, nivolumab, duvalizumab, atezolizumab, etc.), Monoclonal antibody(bevacizumab, cetuximab, trastuzumab, TDM1, etc.), Tyrosine Kinase Inhibitor(TKI)(gefitinib, osimertinib, crizotinib, pyrotinib, anlotinib, fruquintinib, etc.).

### Statistical analysis

Statistical analyses were performed by R software (version 3.3.2). Continuous variables were categorized or dichotomized for the analyses. Chi-square test and two-tailed test were used for categorical variables and continuous variables respectively. Univariable analysis was performed to identify potential predictors of thrombocytopenia. Factors shown to be significant predictors in univariate analysis (*P* < 0.05) were brought forward to a multivariate analysis by a backward stepwise procedure.

Nomogram was used to display the clinical prediction model which was constructed based on the results of multivariate analysis for CIT using the package of rms in R software. The predictive accuracy of the final model was quantified using discrimination and calibration measures. The discrimination and calibration of the model were evaluated using the area under the receiver operating characteristic (ROC) curve. A bootstrapping technique was applied using 1000 random data sets (validation set) generated from the original data.

### Studies involving animal subjects

Generated Statement: No animal studies are presented in this manuscript.

### Studies involving human subjects

Generated Statement: The studies involving human participants were reviewed and approved by Medical Ethics Committee of Affiliated Jinhua Hospital, Zhejiang University School of Medicine (Zhejiang, China). The patients/participants provided their written informed consent to participate in this study.

### Inclusion of identifiable human data

Generated Statement: No potentially identifiable human images or data is presented in this study.

## Result

### Risk factors of CIT based on patients

**Figure 1:**
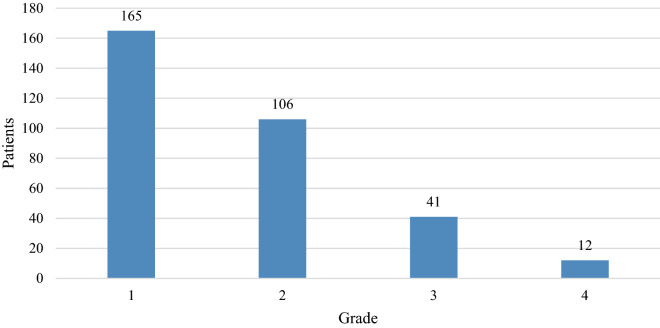
Incidences of grade 1 ~ 4 CIT based on patients.

Initially, we identified 1540 patients with solid tumors receiving chemotherapy during the study period, of which 883 (57.30%) were males and 658 (42.70%) were females. The detailed patients’ demographic and clinical characteristics were summarized in Table 1. Thrombocytopenia occurred in 324 patients, resulting in a frequency of 21.03%. The 5 most common types of cancers are (in the order of frequency) cervix (8, 33.33%), gastric (32, 31.07%), bladder (8, 28.57%), biliary systemic (8, 27.59%), and ovarian (10, 27.03%). Incidences of grade 1, 2, 3, and 4 thrombocytopenia in patients with solid tumors was 50.9%, 32.7%, 12.7%, 3.7%, respectively (Fig. 1). No bleeding was found in the patients.

**Table 1:** Clinical characteristics based on patients.

Characteristic		Total	PLT. low (%)	PLT. normal (%)	*P* value
		1541	324 (21.03)	1217 (78.97)	
Gender					0.437
	Male	883	179 (20.27)	704 (79.73)	
	Female	658	145 (22.04)	513 (77.96)	
Age					0.099
	< 40	47	8 (17.02)	39 (82.98)	
	40–60	575	106 (18.43)	469 (81.57)	
	> 60	919	210 (22.85)	709 (77.15)	
Tumor site					0.049
	Bladder	28	8 (28.57)	20 (71.43)	
	Breast	157	29 (18.47)	128 (81.53)	
	Cervix	24	8 (33.33)	16 (66.67)	
	Crc	514	110 (21.4)	404 (78.6)	
	Easophage	47	9 (19.15)	38 (80.85)	
	Biliary systemic	29	8 (27.59)	21 (72.41)	
	Gastric	103	32 (31.07)	71 (68.93)	
	Lung	299	42 (14.05)	257 (85.95)	
	Ovarian	37	10 (27.03)	27 (72.97)	
	Pancrease	51	11 (21.57)	40 (78.43)	
	mpc	84	18 (21.43)	66 (78.57)	
	Other	139	33 (23.74)	106 (76.26)	
	Unknown	29	6 (20.69)	23 (79.31)	
NRS2002					0.151
	< 4	1226	248 (20.23)	978 (79.77)	
	> 4	315	76 (24.13)	239 (75.87)	
BMI (kg/m2)					0.921
	Low	179	39 (21.79)	140 (78.21)	
	Normal	1252	263 (21.01)	989 (78.99)	
	High	74	16 (21.62)	58 (78.38)	
	Unknown	36	6 (16.67)	30 (83.33)	
PG–SGA					0.017
	A	516	114 (22.09)	402 (77.91)	
	B	253	60 (23.72)	193 (76.28)	
	C	72	23 (31.94)	49 (68.06)	
	unknown	700	127 (18.14)	573 (81.86)	
AST (U/L)					< 0.001
	High	284	109 (38.38)	175 (61.62)	
	Normal	867	154 (17.76)	713 (82.24)	
	Unknown	390	61 (15.64)	329 (84.36)	
Tbil (umol/L)					< 0.001
	High	93	43 (46.24)	50 (53.76)	
	Normal	1333	260 (19.5)	1073 (80.5)	
	Unknown	115	21 (18.26)	94 (81.74)	
ALT (U/L)					0.133
	High	205	54 (26.34)	151 (73.66)	
	Normal	1293	261 (20.19)	1032 (79.81)	
	Unknown	43	9 (20.93)	34 (79.07)	
ALB (g/L)					< 0.001
	Low	52	21 (40.38)	31 (59.62)	
	Normal	560	89 (15.89)	471 (84.11)	
	Unknown	929	214 (23.04)	715 (76.96)	
PLT (*10^9/L)					< 0.001
	Low	324	324 (100)	0 (0)	
	Normal	1217	0 (0)	1217 (100)	
WBC(*10^9/L)					< 0.001
	Low	290	90 (31.03)	200 (68.97)	
	Normal	1251	234 (18.71)	1017 (81.29)	
HB (g/L)					< 0.001
	Low	675	227 (33.63)	448 (66.37)	
	Normal	866	97 (11.2)	769 (88.8)	
CRP (mg/dL)					0.003
	High	308	70 (22.73)	238 (77.27)	
	Normal	320	45 (14.06)	275 (85.94)	
	Unknown	913	209 (22.89)	704 (77.11)	
Liver metastasis					< 0.001
	Yes	477	131 (27.46)	346 (72.54)	
	No	1064	193 (18.14)	871 (81.86)	
Line of treatment					< 0.001
	1	1059	163 (15.39)	896 (84.61)	
	2	152	44 (28.95)	108 (71.05)	
	3	17	8 (47.06)	9 (52.94)	
	4	7	3 (42.86)	4 (57.14)

**Figure 2:**
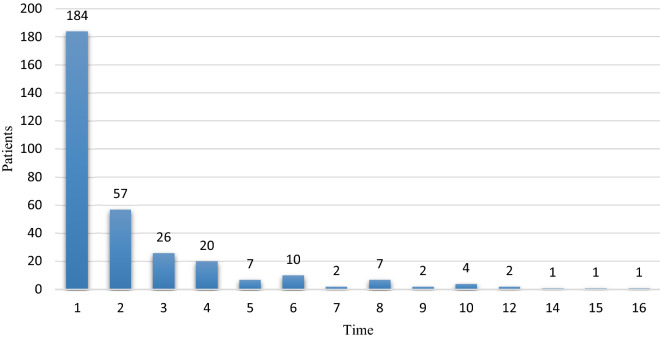
The number and time of occurrence of CIT based on patients.

During the follow-up, CIT only happened once for 189 (58.33%) patients, but 135(41.67%) patients experienced repeated CIT. In the analysis of the number and time of occurrence of CIT, we found that one patient had 16 times of CIT during the anti-tumor treatment, and 9 (2.78%) patients had more than 10 times of CIT (Fig. 2). CIT first appeared when the patient received 5 cycles of chemotherapy. And the PLT decreased to the lowest during the fourth cycle. The lowest count of PLT in 66 (41.8%) patients appeared after the last cycle of chemotherapy. Chemotherapy cycle delay/dose was examined in 81 cancer patients (349 cycles) with complete chemotherapy dose information in the database included myelosuppression, infection, fatigue, severe electrolyte disturbances and liver function injury, among which 45.7% (37/81) were only caused by thrombocytopenia, and grade 3 thrombocytopenia accounted for 43.2%(16/37). In addition, 8 patients had Grade 4 thrombocytopenia, but none had bleeding. RDI in any cycle of the course was 5.39%.

### Risk factors of CIT based on chemotherapy cycles

**Figure 3:**
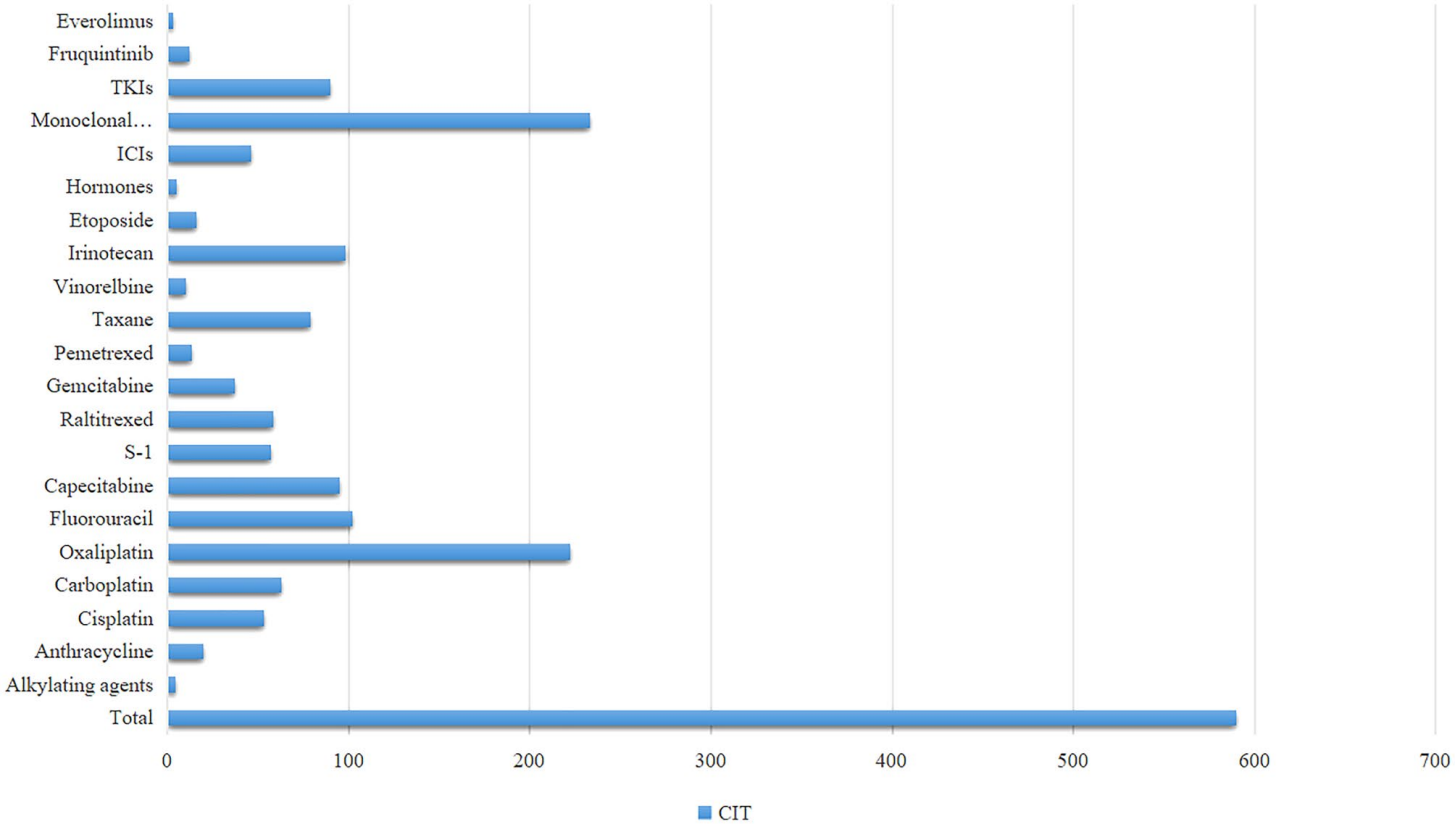
Cumulative number of CIT based on chemotherapy cycles by antineoplastic drugs.

The data were based on 5750 chemotherapy cycles, CIT occurred in 590(10.26%) cycles (Table 2). Patients who received S-1, capecitabine, oxaliplatin, and anthracycline based regimens had a higher incidence of thrombocytopenia (59, 20.34%; 95, 18.13%; 222, 16.78% and 20, 16.53%, respectively) than those who received etoposide, taxanes, gemcitabine, irinotecan and fluorouracil based regimens (15, 7.94%; 79, 7.63%; 37, 7.4%; 98, 7.31% and 102, 6.63%, respectively) (Table 3, Fig. 3). Quite remarkably, TKIs including fruquintinib were significantly related to CIT (*P* = 0.001, *P* = 0.002, respectively). CIT, meanwhile, occurred in patients who have been treated with monoclonal antibody and ICIs (233, 9.28% and 46, 8.85%, respectively).

**Table 2:** Clinical characteristics based on chemotherapy cycles.

Characteristic		Total	PLT. low (%)	PLT. normal (%)	*P* value
		5750	590 (10.26)	5160 (89.74)	
Gender					0.6
	Male	3075	309 (10.05)	2766 (89.95)	
	Female	2675	281 (10.5)	2394 (89.5)	
Age					0.017
	< 40	176	19 (10.8)	157 (89.2)	
	40–60	2282	202 (8.85)	2080 (91.15)	
	> 60	3292	369 (11.21)	2923 (88.79)	
Tumor site					< 0.001
	Bladder	106	5 (4.72)	101 (95.28)	
	Breast	776	45 (5.8)	731 (94.2)	
	Cervix	75	15 (20)	60 (80)	
	Crc	2336	250 (10.7)	2086 (89.3)	
	Easophage	96	7 (7.29)	89 (92.71)	
	Biliary systemic	86	15 (17.44)	71 (82.56)	
	Gastric	303	63 (20.79)	240 (79.21)	
	Lung	955	56 (5.86)	899 (94.14)	
	Ovarian	165	39 (23.64)	126 (76.36)	
	Pancrease	151	12 (7.95)	139 (92.05)	
	mpc	311	26 (8.36)	285 (91.64)	
	Other	330	45 (13.64)	285 (86.36)	
	Unknown	60	12 (20)	48 (80)	
NRS2002					1
	< 4	4911	504 (10.26)	4407 (89.74)	
	> 4	839	86 (10.25)	753 (89.75)	
BMI (kg/m2)					0.247
	Low	536	55 (10.26)	481 (89.74)	
	Normal	4722	488 (10.33)	4234 (89.67)	
	High	355	28 (7.89)	327 (92.11)	
	Unknown	137	19 (13.87)	118 (86.13)	
PG–SGA					0.065
	A	2357	236 (10.01)	2121 (89.99)	
	B	946	118 (12.47)	828 (87.53)	
	C	164	12 (7.32)	152 (92.68)	
	Unknown	2283	224 (9.81)	2059 (90.19)	
AST (U/L)					< 0.001
	High	1065	167 (15.68)	898 (84.32)	
	Normal	3565	325 (9.12)	3240 (90.88)	
	Unknown	1120	98 (8.75)	1022 (91.25)	
ALT (U/L)					0.588
	High	747	73 (9.77)	674 (90.23)	
	Normal	4700	481 (10.23)	4219 (89.77)	
	Unknown	303	36 (11.88)	267 (88.12)	
Tbil (umol/L)					< 0.001
	High	153	30 (19.61)	123 (80.39)	
	Normal	5173	509 (9.84)	4664 (90.16)	
	Unknown	424	51 (12.03)	373 (87.97)	
ALB (g/L)					0.008
	Low	67	14 (20.9)	53 (79.1)	
	Normal	1664	157 (9.44)	1507 (90.56)	
	Unknown	4019	419 (10.43)	3600 (89.57)	
CRP (mg/dL)					0.009
	High	591	64 (10.83)	527 (89.17)	
	Normal	1312	105 (8)	1207 (92)	
	Unknown	3847	421 (10.94)	3426 (89.06)	
PLT (*10^9/L)					< 0.001
	Low	463	285 (61.56)	178 (38.44)	
	Normal	5076	285 (5.61)	4791 (94.39)	
	Unknown	211	20 (9.48)	191 (90.52)	
WBC(*10^9/L)					< 0.001
	Low	993	153 (15.41)	840 (84.59)	
	Normal	4545	417 (9.17)	4128 (90.83)	
	Unknown	212	20 (9.43)	192 (90.57)	
HB (g/L)					< 0.001
	Low	2127	312 (14.67)	1815 (85.33)	
	Normal	3411	258 (7.56)	3153 (92.44)	
	Unknown	212	20 (9.43)	192 (90.57)	
Liver metastasis					0.029
	Yes	2366	268 (11.33)	2098 (88.67)	
	No	3384	322 (9.52)	3062 (90.48)	
Line of treatment	1	4431	399 (9)	4032 (91)	< 0.001
	2	1000	141 (14.1)	859 (85.9)	
	3	243	37 (15.23)	206 (84.77)	
	4	58	8 (13.79)	50 (86.21)	
	5	12	2 (16.67)	10 (83.33)	
	6	4	1 (25)	3 (75)	
	7	2	2 (100)	0 (0)

**Table 3:** Antineoplastic drugs based on chemotherapy cycles.

Antineoplastic drugs		Total	PLT low (%)	*P* value
		5750	590 (10.26)	
Alkylating agents				0.01
	Yes	130	4 (3.08)	
	No	5620	586 (10.43)	
Anthracycline				0.032
	Yes	121	20 (16.53)	
	No	5629	570 (10.13)	
Cisplatin				0.942
	Yes	507	53 (10.45)	
	No	5243	537 (10.24)	
Carboplatin				1
	Yes	614	63 (10.26)	
	No	5136	527 (10.26)	
Oxaliplatin				< 0.001
	Yes	1323	222 (16.78)	
	No	4427	368 (8.31)	
Fluorouracil				< 0.001
	Yes	1538	102 (6.63)	
	No	4212	488 (11.59)	
Capecitabine				< 0.001
	yes	524	95 (18.13)	
	No	5226	495 (9.47)	
S-1				< 0.001
	Yes	283	57 (20.14)	
	No	5467	533 (9.75)	
Raltitrexed				0.323
	Yes	498	58 (11.65)	
	No	5252	532 (10.13)	
Gemcitabine				0.033
	Yes	500	37 (7.4)	
	No	5250	553 (10.53)	
Pemetrexed				0.003
	Yes	272	13 (4.78)	
	No	5478	577 (10.53)	
Taxane				0.002
	Yes	1036	79 (7.63)	
	No	4714	511 (10.84)	
Vinorelbine				0.755
	Yes	112	10 (8.93)	
	No	5638	580 (10.29)	
Irinotecan				< 0.001
	Yes	1341	98 (7.31)	
	No	4409	492 (11.16)	
Etoposide				0.412
	Yes	194	16 (8.25)	
	No	5556	574 (10.33)	
Hormones				0.191
	Yes	90	5 (5.56)	
	No	5660	585 (10.34)	
ICIs				0.299
	Yes	520	46 (8.85)	
	No	5230	544 (10.4)	
Monoclonal antibody				0.034
	Yes	2511	233 (9.28)	
	No	3239	357 (11.02)	
TKIs				0.001
	Yes	632	90 (14.24)	
	No	5118	500 (9.77)	
Fruquintinib				0.002
	Yes	48	12 (25)	
	No	5702	578 (10.14)	
Everolimus				0.582
	Yes	45	3 (6.67)	
	No	5705	587 (10.29)

 Univariate analysis showed that liver metastasis (*P* = 0.029), AST (*P* < 0.001), Tbil (*P* < 0.001), ALB (*P* < 0.001), blood cell counts at the last cycle (PLT, *P* < 0.001; WBC, *P* < 0.001; HB, *P* < 0.001), and CRP (*P* = 0.05) were associated with CIT of patients with solid tumors. The results also showed all chemotherapeutic agents based regimens were closely related to CIT (*P* < 0.05). According to multivariate Logistic regression analysis, it was noticed that AST, Tbil, blood cell counts at the last cycle, oxaliplatin, alkylating agents, pemetrexed, fluorouracil, capecitabine, anthracycline, TKI excluding fruquintinib, and taxane were significantly associated with CIT (Table 4). Considering that patients may have repeated thrombocytopenia during chemotherapy, the probability of CIT may further increase with the increase of chemotherapy times, and different patients have different bone marrow tolerance to the same chemotherapy regimen, balancing the influence of chemotherapy times and chemotherapy regimen, this study is based on further statistical analysis of patients. Based on the original data, we have resubmitted the *appendix*.

**Table 4:** Univariate analysis, multivariate analysis, and factors for building the prediction model based on chemotherapy cycles.

Variables	Univariate analysis		Multivariate analysis	
	HR (95%CI)	*P*	HR (95%CI)	*P*
Gender				
Male	1	–	1	–
Female	1.05 (0.89–1.25)	0.57	1.03 (0.83–1.27)	0.81
Age				
< 40	1		1	–
40–60	0.8 (0.49–1.32)	0.39	0.7 3(0.44–1.3)	0.27
> 60	1.04 (0.64–1.7)	0.87	0.88 (0.53–1.56)	0.65
Tumor site				
Unknown	1	–	1	–
Bladder	0.2(0.07–0.59)	< 0.001	0.23 (0.07–0.68)	0.01
Breast	0.25 (0.12–0.5)	< 0.001	0.24 (0.12–0.54)	< 0.001
Cervix	1 (0.43–2.34)	1	1.23 (0.49–3.1)	0.66
Colorectal	0.48 (0.25–0.91)	0.03	0.42 (0.22–0.89)	0.02
Esophagus	0.31 (0.12–0.85)	0.02	0.44 (0.15–1.23)	0.13
Biliary	0.85 (0.36–1.96)	0.7	0.58 (0.22–1.52)	0.26
Gastric	1.05 (0.53–2.09)	0.89	0.55 (0.25–1.28)	0.15
Lung	0.25 (0.13–0.5)	< 0.001	0.35 (0.17–0.76)	0.01
Ovarian	0.25 (0.13–0.5)	< 0.001	1.71 (0.78–3.93)	0.19
Pancreas	0.36 (0.17–0.77)	0.01	0.41 (0.16–1.06)	0.06
mpc	0.63 (0.31–1.28)	0.2	0.35 (0.16–0.8)	0.01
Other	1.24 (0.6–2.56)	0.56	0.96 (0.46–2.12)	0.92
Liver metastases				
No	1	–	1	–
Yes	1.21 (1.02–1.44)	0.03	1.02 (0.84–1.24)	0.83
WBC				
Normal	1	–	1	–
Low	1.8 (1.48–2.2)	< 0.001	1.89 (1.52–2.35)	< 0.001
Hb				
Normal	1	–	1	–
Low	2.1 (1.76–2.5)	< 0.001	1.94 (1.6–2.36)	< 0.001
Unknown	1.27 (0.79–2.05)	0.32	0.87 (0.43–1.77)	0.71
CRP				
Normal	1	–	1	–
High	1.4 (1.01–1.94)	0.05	1.06 (0.74–1.5)	0.76
Unknown	1.41 (1.13–1.77)	< 0.001	1.31 (1.03–1.67)	0.03
Tbil				
Normal	1	–	1	–
High	2.23 (1.48–3.37)	< 0.001	1.76 (1.1–2.76)	0.02
Unknown	1.25 (0.92–1.7)	0.15	1.57 (0.83–2.81)	0.15
ALB				
Normal	1	–	1	–
Low	2.54 (1.38–4.67)	< 0.001	1.82 (0.91–3.44)	0.08
Unknown	1.12 (0.92–1.35)	0.26	1.08 (0.88–1.34)	0.46
AST				
Normal	1	–	1	–
High	1.85 (1.52–2.27)	< 0.001	1.89 (1.46–2.43)	< 0.001
Unknown	0.96 (0.75–1.21)	0.71	0.76 (0.54–1.04)	0.1
ALT				
Normal	1	–	1	–
High	0.95 (0.73–1.23)	0.7	0.74 (0.54–1.01)	0.06
Unknown	1.18 (0.82–1.7)	0.36	1.64 (0.74–3.69)	0.22
Oxaliplatin				
No	1	–	1	–
Yes	2.22 (1.86–2.66)	< 0.001	2.57 (1.89–3.49)	< 0.001
Pemetrexed				
No	1	–	1	–
Yes	0.43 (0.24–0.75)	< 0.001	0.5 (0.25–0.92)	0.03
Irinotecan				
No	1	–	1	–
Yes	0.63 (0.5–0.79)	< 0.001	1.23 (0.88–1.72)	0.22
Gemcitabine				
no				
yes	0.68 (0.48–0.96)	0.03	0.81 (0.52–1.24)	0.34
Fluorouracil				
No	1	–	1	–
Yes	0.54 (0.43–0.68)	< 0.001	0.54 (0.39–0.75)	< 0.001
Capecitabine				
No	1	–		
Yes	2.12 (1.66–2.69)	< 0.001	1.45 (1.05–1.99)	0.02
S-1				
No	1	**–**	1	**–**
Yes	2.33 (1.72**–**3.16)	< 0.001	1.1 (0.65**–**1.84)	0.71
Fruquintinib				
No	1	**–**	1	**–**
Yes	2.96 (1.53**–**5.71)	< 0.001	1.93 (0.85**–**4.19)	0.11
Alkylating agents				
No	1	**–**	1	**–**
Yes	0.27 (0.1**–**0.74)	0.01	0.2 (0.06**–**0.54)	< 0.001
Anthracycline				
No	1	**–**	1	**–**
Yes	1.76 (1.08**–**2.86)	0.02	2.17 (1.1**–**4.2)	0.02
TKI				
No	1	**–**	1	**–**
Yes	1.53 (1.2**–**1.95)	< 0.001	1.48 (1.07**–**2.04)	0.02
Taxane				
No	1	**–**	1	
Yes	0.68 (0.53**–**0.87)	< 0.001	0.65 (0.46**–**0.91)	0.01
Line of treatment				
1	1	**–**	1	**–**
2	1.66 (1.35**–**2.04)	< 0.001	1.21 (0.95**–**1.54)	0.12
3	1.82 (1.26**–**2.61)	< 0.001	1.57 (1.03**–**2.36)	0.03
4	1.62 (0.76**–**3.43)	0.21	1.26 (0.52**–**2.7)	0.58
5	2.02 (0.44**–**9.26)	0.36	1.98 (0.29**–**8.13)	0.4
6	3.37 (0.35**–**32.46)	0.29	5.78 (0.27**–**49.7)	0.14

### Nomogram for prediction of CIT

**Figure 4:**
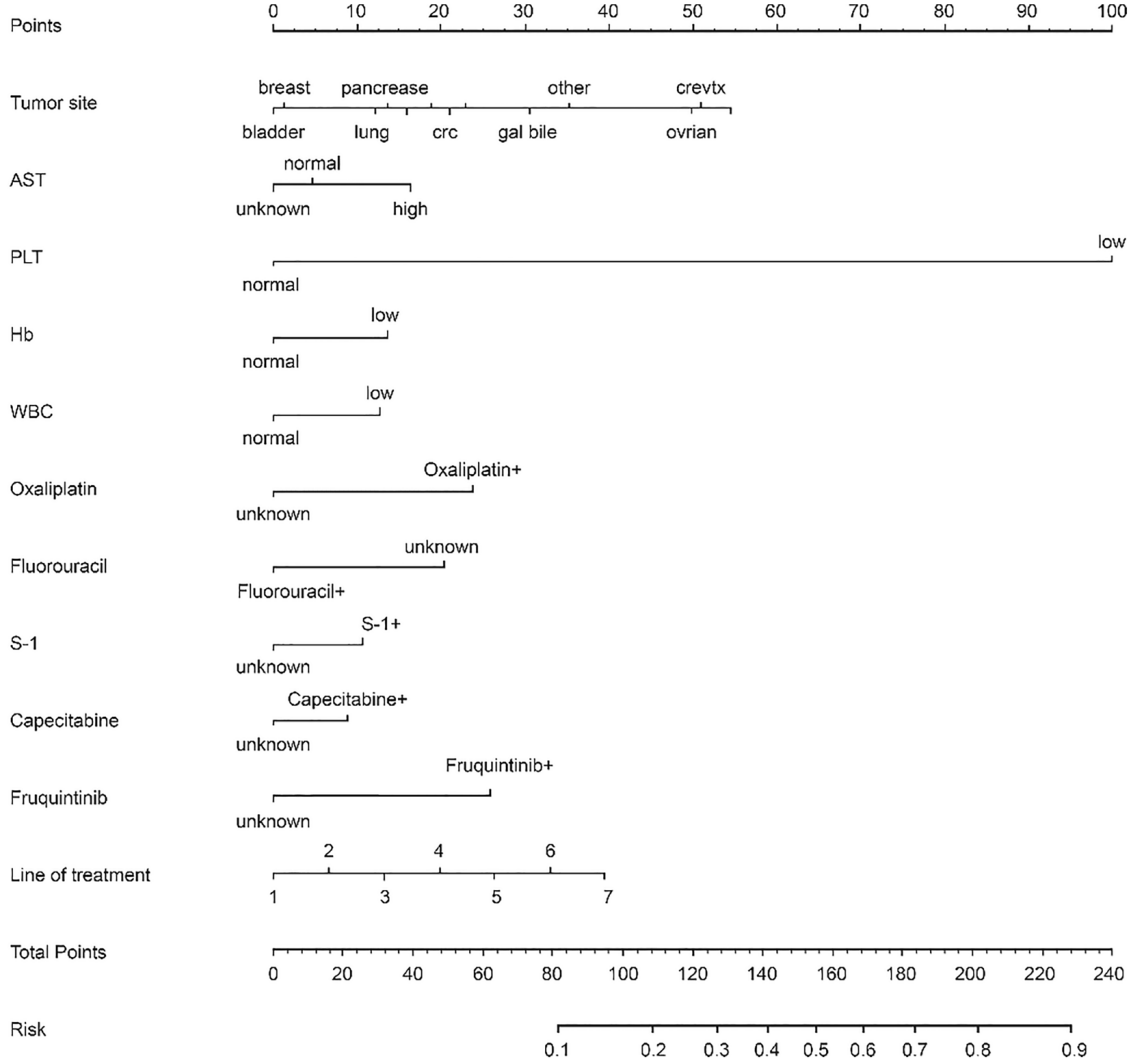
The nomogram model for CIT probability prediction based on chemotherapy cycles.

**Figure 5:**
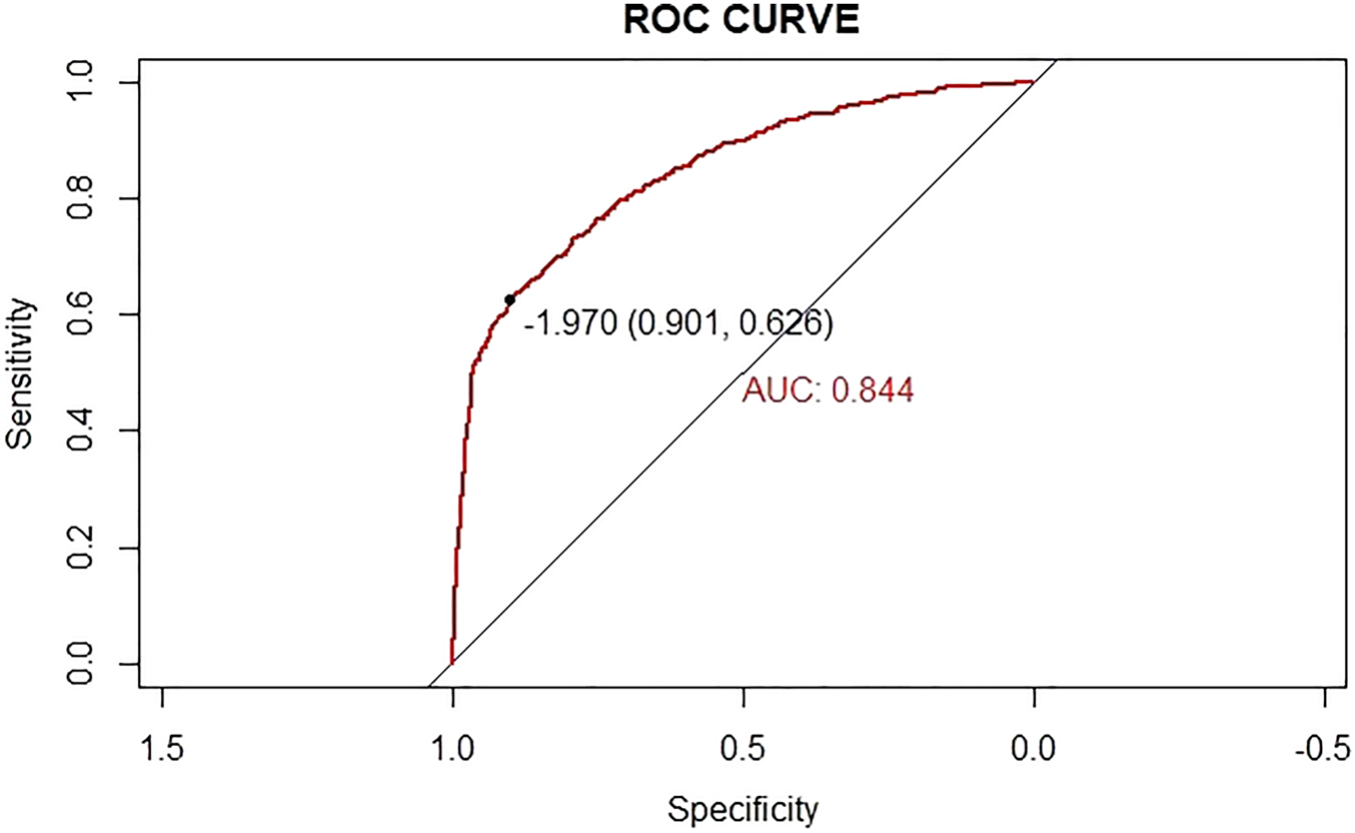
The area under the ROC curve (AUC) value of 0.844 (95% CI: 0.626 to 0.901) indicates that the prediction model has good prediction accuracy.

**Figure 6:**
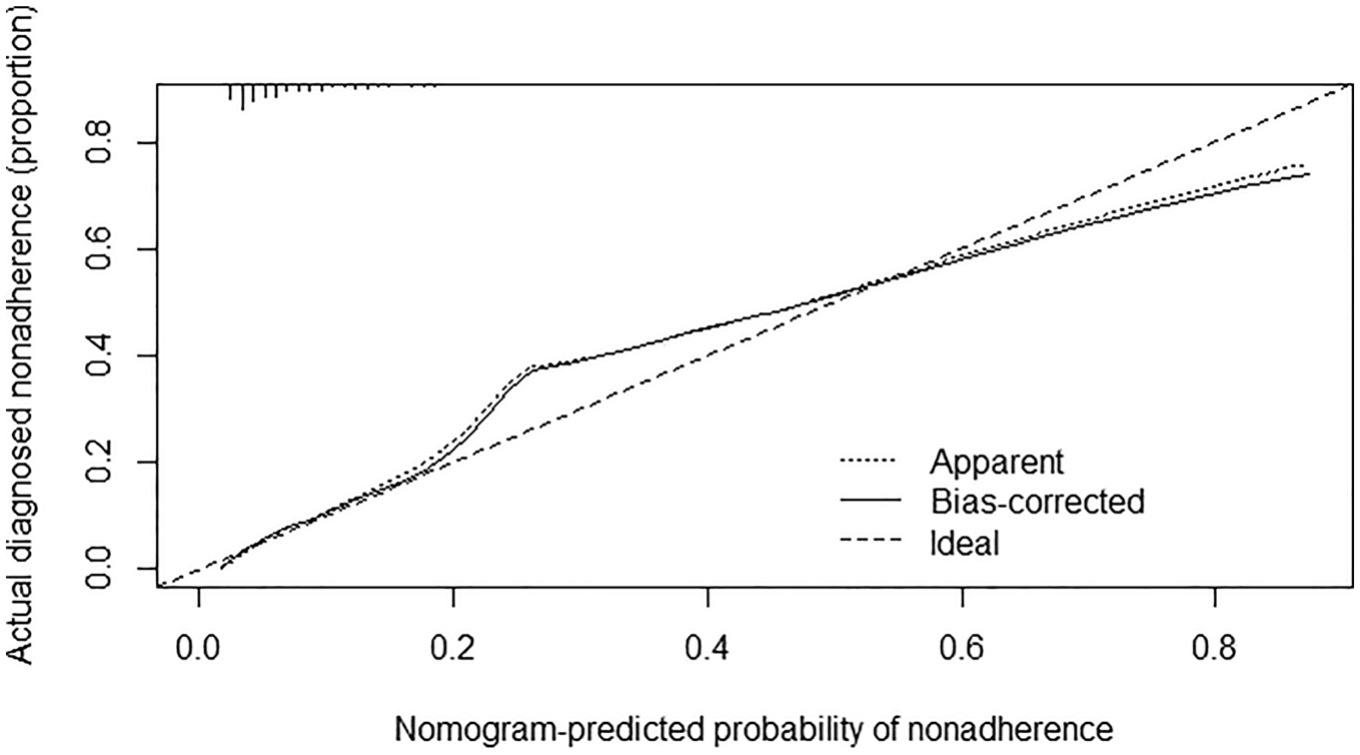
The calibration curve for predicting CIT in the validation set.

In this study, in order to predict the occurrence of CIT, the independent prognostic factors of tumor site, antineoplastic drugs, blood cell counts at the last cycle (RBC, HB, PLT), CRP, liver metastasis and treatment line were used to establish a nomogram model for CIT probability prediction (Fig. 4). A weighted total score calculated from these variables was used to estimate the occurrence of CIT. In these variables, each factor was assigned a number of risk points, which was obtained by drawing a straight line directly upward to the “points” axis from the corresponding value of the prognostic factor. The process was repeated for each prognostic factor. The points obtained for each covariate were summarized, and “Total Point” axis was located from the sum of the risk points. Finally, a vertical line was drawn directly down to the axis that determined the patient’s probability of CIT. The area under the ROC curve (AUC) value of 0.844 (95% CI: 0.626 to 0.901) indicates that the prediction model has good prediction accuracy (Fig. 5). In addition, the calibration plots for the probability of CIT revealed a good match between the prediction of CIT in the nomogram model and the actual observation (Fig. 6).

## Discussion

Patients with solid tumors who received anti-tumor treatment were enrolled in the retrospective cohort. The incidence of CIT in patients with solid tumors was 21.03%, which was 10.26% if based on chemotherapy cycles. The tumor site, treatment line, AST, blood cell counts at the last cycle, oxaliplatin and capecitabine were significantly associated with CIT. In this study, nomogram for prediction of CIT was established on the cohort of more than 1000 patients with solid tumors or lymphomas receiving chemotherapy. The area under the ROC curve (AUC) value of 0.844 indicates that the model has better predictive performance. In addition, the calibration plots for the probability of CIT revealed a good match between the prediction of CIT in the nomogram model and the actual observation.

In our cohort, three common cancers are cervix, gastric and bladder. Unlike Wu's study, the most common cancer was breast cancer (19.5%)^4^. A study explored the incidence and clinical consequences of chemotherapy-induced thrombocytopenia (CIT) among metastatic colorectal cancer (mCRC) patients, and revealed that 37% of mCRC patients experienced CIT^24^. These figures for CRC are significantly higher than our results (21.4%). The incidence of thrombocytopenia was ranging from 25% to 27.5%^25^. This result was slightly less than the incidence of CIT (30.07%) in gastric cancer patients in our study. The incidence of CIT in lung cancer was 12.96%, compared to the treatment caused similar risk of CIT in other clinical studies (ranging from 1 to 23%)^26–30^.

The occurrence of CIT is closely related to chemotherapy regimens and chemotherapy cycles. The incidence of CIT in platinum based regimens is 13.8%. It was lower to another large observational study of 62,072 chemotherapy regimens, the incidence of platinum-associated CIT in solid tumors was 31%^4^. Taking a look at three kinds of platinum, our study found that oxaliplatin was significantly associated with CIT (16.8%), which was similar to previous studies^25,31–33^.

The prevalence of CIT after initiation of chemotherapy was 7.4% in patients treated with gemcitabine-based regimens. Previous studies have shown CIT in more than 20% of patients receiving gemcitabine^27,34,35^. Maarten J. Ten Berg found that the incidence of CIT was 64.4% if patients treated with gemcitabine^36^. It was significantly higher than our study. Furthermore, multivariate analysis in this study showed that gemcitabine was not associated with CIT. A review of real-world data on 215,508 patients with cancer found that the incidence of gemcitabine-containing regimens in hospitalized patients was only 5.5%^20^, which is comparable with the present result. In our study, most chemotherapeutic drugs for solid tumor were involved, and all patients were followed during all chemotherapy cycles. Therefore, the result of this study can reflect those who suffered CIT during chemotherapy in eastern China.

In this study, 5-FU-based regimens did not increase the occurrence of CIT, while capecitabine did the opposite. Previous studies have shown that in gastrointestinal tumors, the incidence of 5-FU-related CIT was from 1.49% to 19.0%^31,32^, which was similar to the results of our study. For capecitabine, however, the incidence of CIT was found to be similar to that of 5-FU^37^. Coincidentally, the incidence of CIT increased in oxaliplatin-based chemotherapy^25,31,33^. Our real world data also confirmed that oxaliplatin was positively correlated with CIT. This suggests that for intractable CIT induced by XELOX, We could try replacing it with FOLFOX. Of course, we could suspend oxaliplatin and continue capecitabine, and the next cycle of combination therapy can be carried out after platelet recovery.

For most chemotherapy regimens, CIT first appeared when the patient received 5 cycles of chemotherapy. Jaime et al. found that patients with solid tumor who underwent chemotherapy developed CIT within 3 months^19^. Moreover, the incidence of CIT went up with the cycles of chemotherapy, which was similar to the results of real-world studies in other regions. In a retrospective study, Bassam et al. found that CIT was positively correlated with the number of chemotherapy cycle. The incidence of CIT was up to 43.8% when the number of chemotherapy cycles ≥ 4^7^.

This study was the first to find that elevated level of AST was associated with CIT. The common drugs which induced acute liver injuries was antineoplastics (15.3%)^38^. The most important being development of chemotherapy associated liver injuries (CALI)^39–42^. The evolution character of CALI was usually accompanied by elevation of ALT, AST, and Tbil. Further research is needed to confirm the intrinsic relationship between them.

At present, only a few studies have established CIT predictive model based on clinical and laboratory variables. In this study, we established a nomogram model for CIT probability prediction in patients with solid tumors (AUC 0.844). In another study, Tanriverdi et al. presented a predictive model based on a single laboratory variable of baseline plasma D-dimer level (an important marker of thrombotic activity) for CIT in patients with sensitivity, specificity, PLR and NLR values of 0.914, 0.897, 3.64 and 0.24^22^. A total of 14 covariates were prospectively assessed as explanatory variables in a cohort of consecutive patients by Razzaghdoust et al^23^. The model performance characteristics of the study include sensitivity 75%, specificity 65.4%, positive likelihood ratio 2.16, and negative likelihood ratio 0.382^23^. The multivariable model exhibited three final predictors for CIT, including body mass index (BMI) < 23 kg/m2 (odds ratio, 2.23; bootstrap *P* = 0.044). However, BMI did not appear to be associated with CIT in our study.

Based on readily available and easily measurable pretreatment factors, our predictive model may help to distinguish a group of patients at high risk for CIT and those who might benefit from prophylactic administration of thrombopoietic growth factors. However, several limitations should be mentioned. CIT events were identified based on the evidence of either thrombocytopenia during hospitalization or documented medical encounters during bleeding rather than through laboratory results, and it may not reflect the overall incidence of CIT owing to lack of PLT count of out-patients. Most of the patients in this study received gemcitabine on day 8 in outpatient department, from whom we could not obtain the result of blood routine test. That may be the main reason for the relatively low incidence of gemcitabine-related CIT in the present study. And it could be partly explained by the fact that platelet data in this study were collected before the start of the next cycle of chemotherapy from those who had passed the myelosuppression phase. In addition, our model was based on medical records of patients in a single center. Since a single population is not fully representative of the whole population, external validation especially in a community setting will be critical to further demonstrate generality and utility of the model.

In conclusion, we assessed the associations of a panel of clinical variables, available in routine clinical practice, with the occurrence of CIT in the cohort of cancer patients with different solid tumors. There were 11 final variables associated with CIT in the nomogram for prediction of CIT. The prediction model could help to identify patients at risk of CIT and complement the clinical management of thrombocytopenia, but further larger studies are required before clinical application.

## Supplementary Information

Supplementary Information 1.

Supplementary Information 2.

Supplementary Information 3.

Supplementary Information 4.

Supplementary Information 5.

## Data Availability

Generated Statement: The raw data in this study were all from the electronic medical record system of hospitalization. All the original data have been strictly reviewed by the study members, and all the data are true, reliable and repeatable. The original contributions presented in the study are included in the article/supplementary material, further inquiries can be directed to the corresponding author/s.
