# Supplementary material for: The predictive model for risk of chemotherapy-induced thrombocytopenia based on antineoplastic drugs for solid tumors in eastern China
Source: Sci Rep. 2023 Feb 23;13:3185. doi: 10.1038/s41598-023-27824-9 (PMC9950128; doi:10.1038/s41598-023-27824-9)
Supplement: Supplementary file 2 — Supplementary Information 2. [file 41598_2023_27824_MOESM2_ESM.zip › Table1.docx]

Table1- Demographics and clinical characteristics of cancer patients.

| **Variables** | **Total** | **PLT.low(%)** | **PLT.normal(%)** | ***P*-value** |
| --- | --- | --- | --- | --- |
|  | 2043 | 419(20.51) | 1624(79.49) |  |
| **Gender** |  |  |  | 0.104 |
| male | 1224 | 236(19.28) | 988(80.72) |  |
| female | 819 | 183(22.34) | 636(77.66) |  |
| **Age** |  |  |  | 0.494 |
| 40-49 | 191 | 31(16.23) | 160(83.77) |  |
| 50-59 | 526 | 108(20.53) | 418(79.47) |  |
| 60-69 | 763 | 165(21.63) | 598(78.37) |  |
| 70-79 | 427 | 89(20.84) | 338(79.16) |  |
| ≥80 | 74 | 17(22.97) | 57(77.03) |  |
| <40 | 62 | 9(14.52) | 53(85.48) |  |
| **Site** |  |  |  | <0.001 |
| bladder | 32 | 9(28.12) | 23(71.88) |  |
| breast | 183 | 34(18.58) | 149(81.42) |  |
| cervix | 27 | 10(37.04) | 17(62.96) |  |
| colorectal | 685 | 140(20.44) | 545(79.56) |  |
| easophage | 76 | 15(19.74) | 61(80.26) |  |
| biliary | 37 | 13(35.14) | 24(64.86) |  |
| gastric | 136 | 45(33.09) | 91(66.91) |  |
| head | 37 | 6(16.22) | 31(83.78) |  |
| lung | 397 | 56(14.11) | 341(85.89) |  |
| ovrian | 41 | 12(29.27) | 29(70.73) |  |
| pancrease | 62 | 14(22.58) | 48(77.42) |  |
| sarcoma | 25 | 4(16) | 21(84) |  |
| mpc | 128 | 24(18.75) | 104(81.25) |  |
| other | 121 | 28(23.14) | 93(76.86) |  |
| unknown | 56 | 9(16.07) | 47(83.93) |  |
| **Liver metastases** |  |  |  | <0.001 |
| yes | 586 | 157(26.79) | 429(73.21) |  |
| unknown | 1457 | 262(17.98) | 1195(82.02) |  |
| **PLT** |  |  |  | <0.001 |
| low | 419 | 419(100) | 0(0) |  |
| normal | 1624 | 0(0) | 1624(100) |  |
| **WBC** |  |  |  | <0.001 |
| low | 490 | 161(32.86) | 329(67.14) |  |
| normal | 1553 | 258(16.61) | 1295(83.39) |  |
| **Hb** |  |  |  | <0.001 |
| low | 853 | 285(33.41) | 568(66.59) |  |
| normal | 1190 | 134(11.26) | 1056(88.74) |  |
| **CRP** |  |  |  | 0.011 |
| high | 408 | 98(24.02) | 310(75.98) |  |
| normal | 451 | 72(15.96) | 379(84.04) |  |
| unknown | 1184 | 249(21.03) | 935(78.97) |  |
| **Tbil** |  |  |  | <0.001 |
| high | 125 | 57(45.6) | 68(54.4) |  |
| normal | 1779 | 335(18.83) | 1444(81.17) |  |
| unknown | 139 | 27(19.42) | 112(80.58) |  |
| **Alb** |  |  |  | <0.001 |
| low | 98 | 39(39.8) | 59(60.2) |  |
| normal | 869 | 142(16.34) | 727(83.66) |  |
| unknown | 1076 | 238(22.12) | 838(77.88) |  |
| **AST** |  |  |  | <0.001 |
| high | 392 | 142(36.22) | 250(63.78) |  |
| normal | 1188 | 205(17.26) | 983(82.74) |  |
| unknown | 463 | 72(15.55) | 391(84.45) |  |
| **ALT** |  |  |  | 0.666 |
| high | 198 | 45(22.73) | 153(77.27) |  |
| normal | 1790 | 364(20.34) | 1426(79.66) |  |
| unknown | 55 | 10(18.18) | 45(81.82) |  |
| **AST/ALT-ratio** | 1.43 [1.07, 1.93] | 1.60 [1.21, 2.20] | 1.38 [1.03, 1.81] | <0.001 |
