# Supplementary material for: The predictive model for risk of chemotherapy-induced thrombocytopenia based on antineoplastic drugs for solid tumors in eastern China
Source: Sci Rep. 2023 Feb 23;13:3185. doi: 10.1038/s41598-023-27824-9 (PMC9950128; doi:10.1038/s41598-023-27824-9)
Supplement: Supplementary file 2 — Supplementary Information 2. [file 41598_2023_27824_MOESM2_ESM.zip › Table2.docx]

**Table2—Clinical Characteristics based on chemotherapy cycles**

| Variables | Total | PLT.low(%) | PLT.normal(%) | *P*-value |
| --- | --- | --- | --- | --- |
|  | 7676 | 768(10.01) | 6908(89.99) |  |
| Gender |  |  |  | 0.515 |
| male | 4208 | 412(9.79) | 3796(90.21) |  |
| female | 3468 | 356(10.27) | 3112(89.73) |  |
| Age |  |  |  | 0.724 |
| 40-49 | 827 | 73(8.83) | 754(91.17) |  |
| 50-59 | 2127 | 211(9.92) | 1916(90.08) |  |
| 60-69 | 2953 | 300(10.16) | 2653(89.84) |  |
| 70-79 | 1397 | 148(10.59) | 1249(89.41) |  |
| ≥80 | 148 | 17(11.49) | 131(88.51) |  |
| <40 | 224 | 19(8.48) | 205(91.52) |  |
| Site |  |  |  | <0.001 |
| appendix | 15 | 1(6.67) | 14(93.33) |  |
| bladder | 128 | 7(5.47) | 121(94.53) |  |
| breast | 981 | 62(6.32) | 919(93.68) |  |
| cervix | 101 | 16(15.84) | 85(84.16) |  |
| colorectal | 3104 | 322(10.37) | 2782(89.63) |  |
| easophage | 153 | 15(9.8) | 138(90.2) |  |
| biliary | 121 | 22(18.18) | 99(81.82) |  |
| gastric | 416 | 91(21.88) | 325(78.12) |  |
| head | 81 | 6(7.41) | 75(92.59) |  |
| liver | 19 | 9(47.37) | 10(52.63) |  |
| lung | 1288 | 74(5.75) | 1214(94.25) |  |
| ovrian | 200 | 41(20.5) | 159(79.5) |  |
| pancrease | 192 | 17(8.85) | 175(91.15) |  |
| sarcoma | 79 | 4(5.06) | 75(94.94) |  |
| mpc | 445 | 34(7.64) | 411(92.36) |  |
| other | 248 | 30(12.1) | 218(87.9) |  |
| unknown | 105 | 17(16.19) | 88(83.81) |  |
| Liver metastases |  |  |  | 0.143 |
| yes | 3055 | 325(10.64) | 2730(89.36) |  |
| unknown | 4621 | 443(9.59) | 4178(90.41) |  |
| PLT |  |  |  | <0.001 |
| low | 609 | 360(59.11) | 249(40.89) |  |
| normal | 6804 | 380(5.58) | 6424(94.42) |  |
| unknown | 263 | 28(10.65) | 235(89.35) |  |
| WBC |  |  |  | <0.001 |
| low | 1698 | 255(15.02) | 1443(84.98) | <0.001 |
| normal | 5714 | 485(8.49) | 5229(91.51) |  |
| unknown | 264 | 28(10.61) | 236(89.39) |  |
| Hb |  |  |  | <0.001 |
| low | 2900 | 405(13.97) | 2495(86.03) |  |
| normal | 4512 | 335(7.42) | 4177(92.58) |  |
| unknown | 264 | 28(10.61) | 236(89.39) |  |
| CRP |  |  |  | 0.020 |
| high | 820 | 77(9.39) | 743(90.61) |  |
| normal | 1825 | 154(8.44) | 1671(91.56) |  |
| unknown | 5031 | 537(10.67) | 4494(89.33) |  |
| Tbil |  |  |  | <0.001 |
| high | 200 | 45(22.5) | 155(77.5) |  |
| normal | 6945 | 661(9.52) | 6284(90.48) |  |
| unknown | 531 | 62(11.68) | 469(88.32) |  |
| Alb |  |  |  | 0.002 |
| low | 147 | 25(17.01) | 122(82.99) |  |
| normal | 3163 | 285(9.01) | 2878(90.99) |  |
| unknown | 4366 | 458(10.49) | 3908(89.51) |  |
| AST |  |  |  | <0.001 |
| high | 1375 | 225(16.36) | 1150(83.64) |  |
| normal | 4816 | 414(8.6) | 4402(91.4) |  |
| unknown | 1485 | 129(8.69) | 1356(91.31) |  |
| ALT |  |  |  | 0.249 |
| high | 627 | 53(8.45) | 574(91.55) |  |
| normal | 6670 | 671(10.06) | 5999(89.94) |  |
| unknown | 379 | 44(11.61) | 335(88.39) |  |
| AST/ALT-ratio | 1.36[1.03, 1.81] | 1.58 [1.20, 2.00] | 1.34 [1.02, 1.78] | <0.001 |
