# Supplementary material for: The predictive model for risk of chemotherapy-induced thrombocytopenia based on antineoplastic drugs for solid tumors in eastern China
Source: Sci Rep. 2023 Feb 23;13:3185. doi: 10.1038/s41598-023-27824-9 (PMC9950128; doi:10.1038/s41598-023-27824-9)
Supplement: Supplementary file 2 — Supplementary Information 2. [file 41598_2023_27824_MOESM2_ESM.zip › Table3.docx]

**Table3- Univariate and multivariate analysis**

| Variables | Univariate analysis | | Multivariate analysis | |
| --- | --- | --- | --- | --- |
|  | HR (95%CI) | *P* | HR (95%CI) | *P* |
| Gender |  |  |  |  |
| male | 1 | - | 1 | - |
| female | 1.05(0.91-1.22) | 0.49 | 0.89(0.71-1.11) | 0.3 |
| Age |  |  |  |  |
| <40 | 1 |  | 1 | - |
| 40-49 | 1.04(0.62-1.77) | 0.87 | 1.03(0.52-2.14) | 0.93 |
| 50-59 | 1.19(0.73-1.94) | 0.49 | 1.17(0.62-2.32) | 0.64 |
| 60-69 | 1.22(0.75-1.98) | 0.42 | 1.19(0.64-2.36) | 0.6 |
| 70-79 | 1.28(0.78-2.11) | 0.34 | 1.05(0.55-2.13) | 0.88 |
| ≥80 | 1.4(0.7-2.79) | 0.34 | 1.12(0.30-3.01) | 0.83 |
| Site |  |  |  |  |
| unknown | 1 | - | 1 | - |
| appendix | 0.37（0.05-3） | 0.35 | 0.79(0.04-5.4) | 0.84 |
| bladder | 0.3（0.12-0.75） | 0.01 | 0.42(0.13-3.17) | 0.15 |
| breast | 0.35（0.2-0.62） | <0.001 | 0.29(0.12-0.76) | 0.01 |
| cervix | 0.97（0.46-2.05） | 0.95 | 1.06(0.36-3.23) | 0.92 |
| colorectal | 0.6（0.35-1.02） | 0.06 | 0.62(0.28-1.55) | 0.27 |
| easophage | 0.56（0.27-1.18） | 0.13 | 0.5(0.18-1.5) | 0.2 |
| biliary | 1.15（0.57-2.3) | 0.69 | 0.83(0.29-2.49) | 0.74 |
| gastric | 1.45(0.82-2.56) | 0.2 | 0.98(0.42-2.54) | 0.97 |
| head | 0.41(0.16-1.1) | 0.08 | 0.31(0.06-1.28) | 0.12 |
| liver | 4.66(1.65-13.17) | <0.001 | 2.82(0.64-12.42) | 0.17 |
| lung | 0.32(0.18-0.56) | <0.001 | 0.4(0.17-1.03) | 0.04 |
| mpc | 0.43(0.23-0.8) | 0.01 | 0.43(0.18-1.17) | 0.08 |
| other | 0.71(0.37-1.36) | 0.3 | 0.66(0.26-1.8) | 0.39 |
| ovrian | 1.33(0.72-2.49) | 0.36 | 1.26(0.5-3.43) | 0.63 |
| pancrease | 0.5(0.24-1.03) | 0.06 | 0.52(0.19-1.51) | 0.22 |
| sarcoma | 0.28(0.09-0.86) | 0.03 | 0.56(0.13-2.02) | 0.39 |
| Liver metastases |  |  |  |  |
| no | 1 | - | 1 | - |
| yes | 1.12(0.97-1.31) | 0.13 | 0.94(0.75-1.16) | 0.54 |
| PLT |  |  |  |  |
| normal |  | - |  | - |
| low | 24.44(20.17-29.61) | <0.001 | 17.78(14.27-22.19) | <0.001 |
| WBC |  |  |  |  |
| normal | 1 | - | 1 | - |
| high | 1.91(1.62-2.24) | <0.001 | 1.49(1.19-1.84) | <0.001 |
| Hb |  |  |  |  |
| normal | 1 | - | 1 | - |
| low | 2.02(1.74-2.36) | <0.001 | 1.43(1.16-1.76) | <0.001 |
| CRP |  |  |  |  |
| normal | 1 | - | 1 | - |
| high | 1.12(0.84-1.5) | 0.42 | 0.86(0.58-1.24) | 0.44 |
| unknown | 1.3(1.07-1.56) | 0.01 | 1.06(0.84-1.35) | 0.62 |
| Tbil |  |  |  |  |
| normal | 1 | - | 1 | - |
| high | 2.76(1.96-3.88) | <0.001 | 1.68(1.01-2.71) | 0.04 |
| unknown | 1.26(0.95-1.66) | 0.11 | 1.15(0.27-3.65) | 0.84 |
| Alb |  |  |  |  |
| normal | 1 | - | 1 | - |
| low | 2.07(1.32-3.24) | <0.001 | 1.23(0.66-2.18) | 0.5 |
| unknown | 1.18（1.01-1.38） | 0.03 | 1.07(0.87-1.32) | 0.51 |
| AST |  |  |  |  |
| normal | 1 | - | 1 | - |
| high | 2.08(1.75-2.48) | <0.001 | 1.69(1.33-2.14) | <0.001 |
| ALT |  |  |  |  |
| normal | 1 | - | 1 | - |
| high | 0.83(0.62-1.11) | 0.2 | 0.77(0.51-1.15) | 0.2 |
| AST/ALT-ratio | 1.42(1.28-1.56) | <0.001 | 1.23(1.08-1.41) | <0.001 |
